# Automated Clustering Technique (ACT) for Early Onset Scoliosis: A preliminary report

**DOI:** 10.1007/s43390-022-00634-1

**Published:** 2023-01-26

**Authors:** Girish Viraraghavan, Patrick J. Cahill, Michael G. Vitale, Brendan A. Williams, Pediatric Spine Study Group, Sriram Balasubramanian

**Affiliations:** 1 School of Biomedical Engineering, Science and Health Systems, Drexel University, 3141 Chestnut Street, Bossone 718, Philadelphia, PA 19104 USA; 2 Division of Orthopaedics, The Children’s Hospital of Philadelphia, Philadelphia, PA USA; 3 Division of Pediatric Orthopaedic Surgery, Department of Orthopaedic Surgery, Morgan Stanley Children’s Hospital of New York-Presbyterian, Columbia University Medical Center, Columbia University, New York, NY USA; 4 Valley Forge, PA USA

**Keywords:** Early onset scolosis, Classification, Fuzzy C-means, Automated, Clustering

## Abstract

**Purpose:**

While the C-EOS system helps organize and classify Early Onset Scoliosis (EOS) pathology, it is not data-driven and does not help achieve consensus for surgical treatment. The current study aims to create an automated method to cluster EOS patients based on pre-operative clinical indices.

**Methods:**

A total of 1114 EOS patients were used for the study, with the following distribution by etiology: congenital (240), idiopathic (217), neuromuscular (417), syndromic (240). Pre-operative clinical indices used for clustering were age, major curve (Cobb) angle, kyphosis, number of levels involved in a major curve (Cobb angle) and kyphosis along with deformity index (defined as the ratio of major Cobb angle and kyphosis). Fuzzy C-means clustering was performed for each etiology individually, with one-way ANOVA performed to assess statistical significance (*p* < 0.05).

**Results:**

The automated clustering method resulted in three clusters per etiology as the optimal number based on the highest average membership values. Statistical analyses showed that the clusters were significantly different for all the clinical indices within and between etiologies. Link to the ACT-EOS web application: https://biomed.drexel.edu/labs/obl/toolkits/act-eos-application.

**Conclusion:**

An automated method to cluster EOS patients based on pre-operative clinical indices was developed identifying three unique, data-driven subgroups for each C-EOS etiology category. Adoption of such an automated clustering framework can help improve the standardization of clinical decision-making for EOS.

## Introduction

Early onset scoliosis (EOS) is a progressive spine deformity defined by an onset of spinal curvature greater than 10 degrees in children under 10 years of age [1, 2]. EOS is believed to account for nearly 10% of all pediatric scoliosis cases, with the true prevalence unknown [3, 4]. While EOS encompasses different etiologies with a variable natural history, the resulting progressive spine and rib cage deformity that can very likely lead to thoracic insufficiency syndrome which is characterized by reduced lung development and growth [4–7]. To help better understand clinical presentations in the heterogeneous EOS patient population, the Classification of EOS (C-EOS) was developed by an expert committee of spine surgeons to group patients using pre-operative clinical indices such as age, etiology (congenital, idiopathic, neuromuscular, syndromic), major curve (Cobb) angle (< 20°, 20–50°, > 50- < 90°, > 90°) and kyphosis (< 20°, 20–50°, > 50°) [8]. The C-EOS system helps assign a patient to 1 of 48 subgroups based on the aforementioned clinical indices. In 12 of these subgroups, patients have < 20° of major curve (Cobb) angle group and rarely need surgical intervention due to the low extent of the deformity.

C-EOS is the only available classification system to group EOS patients based on the pre-operative clinical indices. While the system is both reliable and accurate, the cut-offs for major curve (Cobb) angle and kyphosis are not based on a data-driven approach [9]. In addition, the 48 subgroups in C-EOS limit meaningful analysis due to a small number of patients in each subgroup, thereby making it difficult to correlate interventions with outcomes [10]. While C-EOS has helped establish a standardized method to communicate different aspects of EOS deformity, it is not widely used like the Lenke classification system for Adolescent Idiopathic Scoliosis (AIS) to help guide surgical treatment [11, 12].

Currently, there is limited consensus among surgeons on treatment selection with variations in the timing of surgery, selection of treatment modality, and levels involved in the instrumentation [13–16]. Management decisions are guided more by a clinician’s experience and training, complicating outcome comparisons between institutions. This is in part due to C-EOS system not providing guidance on treatment decision-making. Clustering methods have been used to detect clinically relevant curve patterns for the AIS population using pre-operative clinical indices and a few studies have even used a data-driven approach to cluster such patients [17–21]. Such automated clustering methods and analysis have yet to be attempted for EOS.

Due to the need for data-driven grouping of the heterogenous EOS patient population, the objective of this study is to use machine learning-based clustering methods to generate a limited number of automated, meaningful subgroups based on pre-operative clinical indices of EOS patients. Such a framework can be used along with the existing C-EOS system as the foundation to build a novel automated classification system informed by patient data to provide standardized guidance for surgical interventions for EOS.

## Methods

### Patient data

After institutional review board approval, EOS patients were retrospectively selected from the prospectively maintained database of the Pediatric Spine Study Group (PSSG) over a 20-year period (1995–2015). For all subjects, pre-operative PA, and lateral radiographs along with measurement of pre-operative clinical indices were obtained. For patients with multiple clinical visits, only the clinical indices from the first visit were considered. Subjects with missing clinical indices were excluded, which resulted in a total of 1170 subjects (congenital (*n* = 259), idiopathic (*n* = 224), neuromuscular (*n* = 440), and syndromic (*n* = 247)) being included for the automated clustering analyses.

### Clinical indices measurement and automated clustering method

Pre-operative clinical indices used for clustering were age, major curve (Cobb) angle, kyphosis, number of levels involved in a major curve (Cobb angle) and kyphosis along with deformity index (defined as the ratio of major Cobb angle and kyphosis.

Fuzzy C-means clustering algorithm is an automated method to group data into a specific number of clusters provided by the user. The algorithm works by initially finding cluster centroids based on the data, after which every datapoint is assigned a membership value that denotes how close it is to the cluster centroid. Based on these membership values, datapoints are clustered together [22]. In the current study, a Fuzzy C-means clustering algorithm was used to cluster EOS patients for each etiology based on age, major curve (Cobb) angle, kyphosis, number of levels involved in the major curve, number of levels involved in kyphosis and deformity index. Hence, four cluster analyses were performed for each of the four EOS etiologies namely congenital, idiopathic, neuromuscular, and syndromic. One of the advantages of the fuzzy c-means clustering algorithm is that it considers the heterogeneity of the dataset and is able to identify outliers using the membership values.

For this study, the optimal number of clusters for each etiology was decided based on the average membership values of the clusters. Different number of clusters were used to perform the clustering analysis, and the one that resulted in the highest average membership value was selected as the optimal number.

### Clustering evaluation metric

The membership values calculated during the fuzzy C-means analyses describe how close a datapoint is to the calculated cluster centroid, hence offering the basis to detect outliers. These membership values help quantify the certainty (i.e. accuracy) of a data point belonging to a particular cluster. Since these values provide accuracy on a data point and its cluster center, these can also be used a metric to determine outliers by defining a lower bound. For the current study, a membership value of 0.6 was used as the lower bound with values below this deemed as outliers. Hence, subjects who had clinical indices with membership values lower than 0.6 were considered as not belonging to a cluster and were excluded from the clustering analyses.

### Statistical analysis

MATLAB (v2020b, The MathWorks Inc, Natick, MA) was used to perform statistical analyses. Average and standard deviation values for each cluster within an etiology were computed and compared with each other using a one-way ANOVA (< 0.01), to detect differences between the clusters. Furthermore, Tukey’s post-hoc analysis was performed to detect differences between the clusters of all the etiologies.

## Results

For fuzzy-C-means, based on the average membership values, three clusters per etiology resulted in the highest membership values. Out of 1170 eligible subjects, a total of 56 subjects (congenital (*n* = 19 out of 259), idiopathic (*n* = 7 out of 224), neuromuscular (*n* = 23 out of 440), and syndromic (*n* = 7 out of 247)) were excluded from the analyses due to their low membership values (i.e. < 0.6), resulting in a total of 1114 subjects being included for the final analyses.

**Fig. 1:**
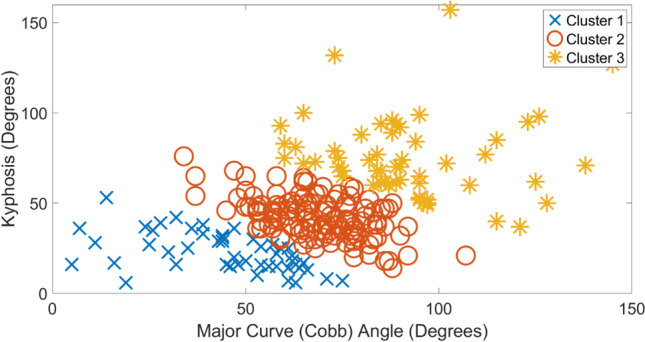
Kyphosis vs major curve (Cobb) angle Congenital EOS etiology

**Fig. 2:**
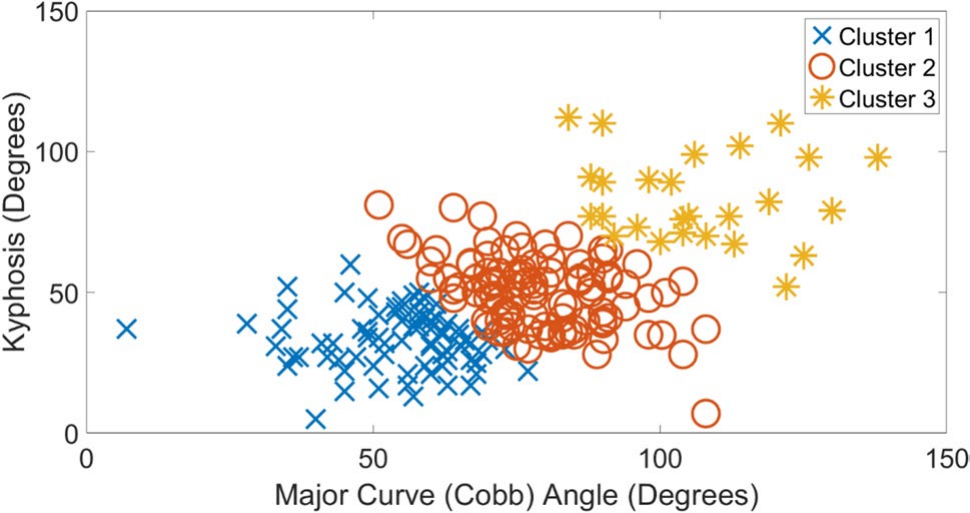
Kyphosis vs major curve (Cobb) angle Idiopathic EOS etiology

**Fig. 3:**
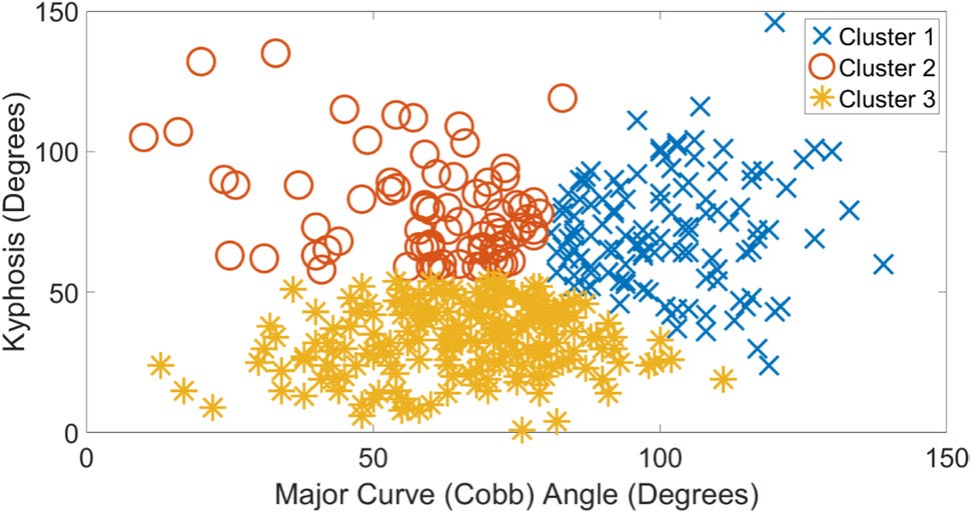
Kyphosis vs major curve (Cobb) angle Neuromuscular EOS etiology

**Fig. 4:**
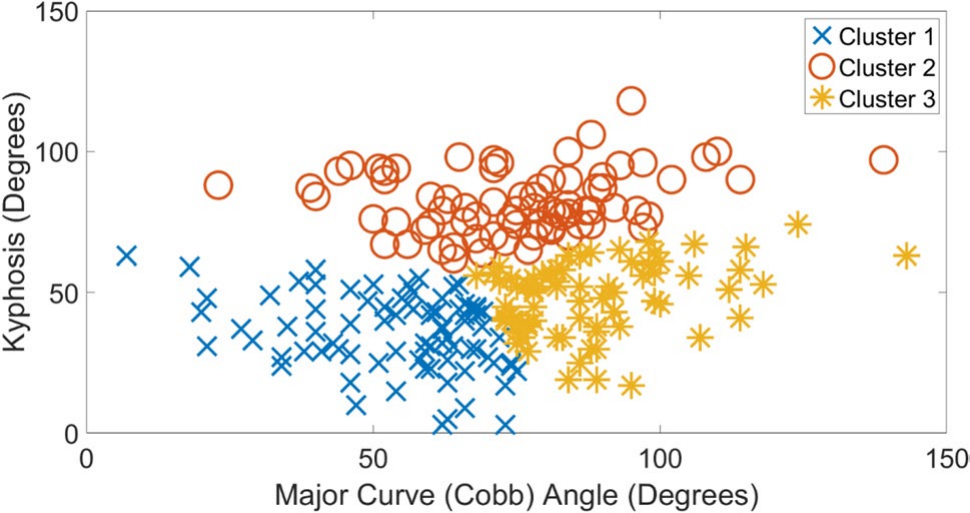
Kyphosis vs mjor curve (Cobb) angle Syndromic EOS etiology

Tables 1, 2, 3, 4 show the average and standard deviation values of clinical indices for each etiology along with exemplar radiographic images. In each table, clusters 1–3 are arranged in ascending order of average magnitude of major curve (Cobb) angle. To best visualize the clusters, scatter plots of kyphosis versus major curve (Cobb) for each etiology are shown in Figs. 1, 2, 3, 4, respectively. For each etiology, one-way ANOVA showed that the clinical indices for any cluster were significantly different (*p* < 0.01) from other clusters. Link to the ACT-EOS web application: https://biomed.drexel.edu/labs/obl/toolkits/act-eos-application.

**Table 1:**
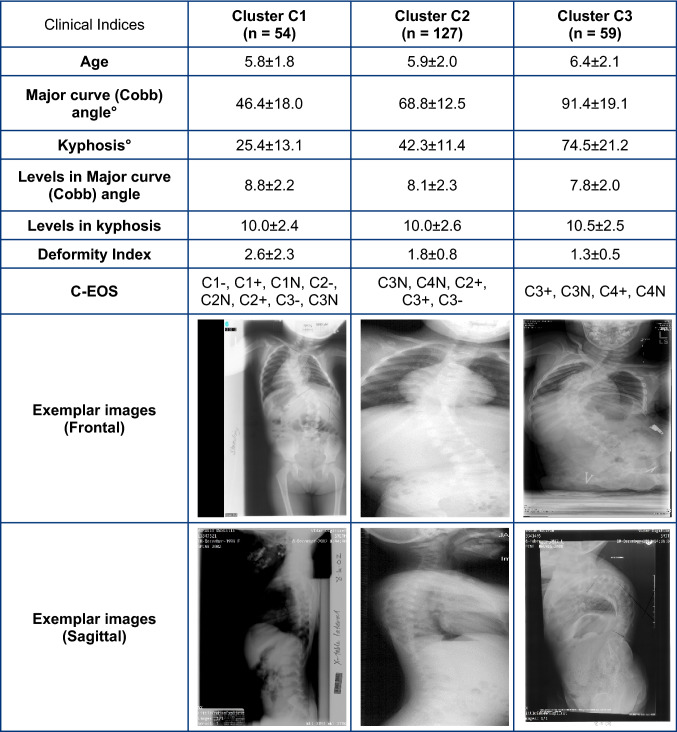
Average and standard deviations values of clinical indices along with exemplar radiographic images for Congenital EOS clusters

**Table 2:**
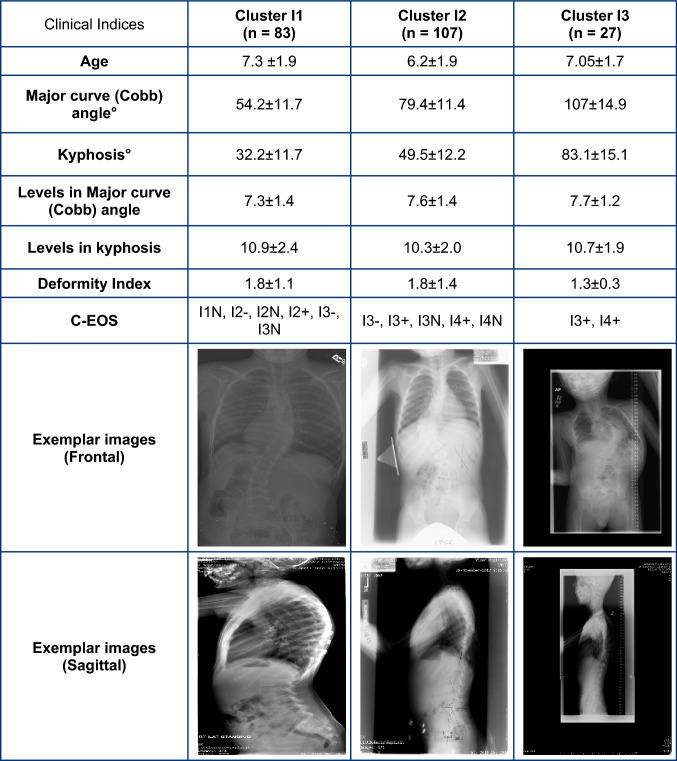
Average and standard deviations values of clinical indices along with exemplar radiographic images for Idiopathic EOS clusters

**Table 3:**
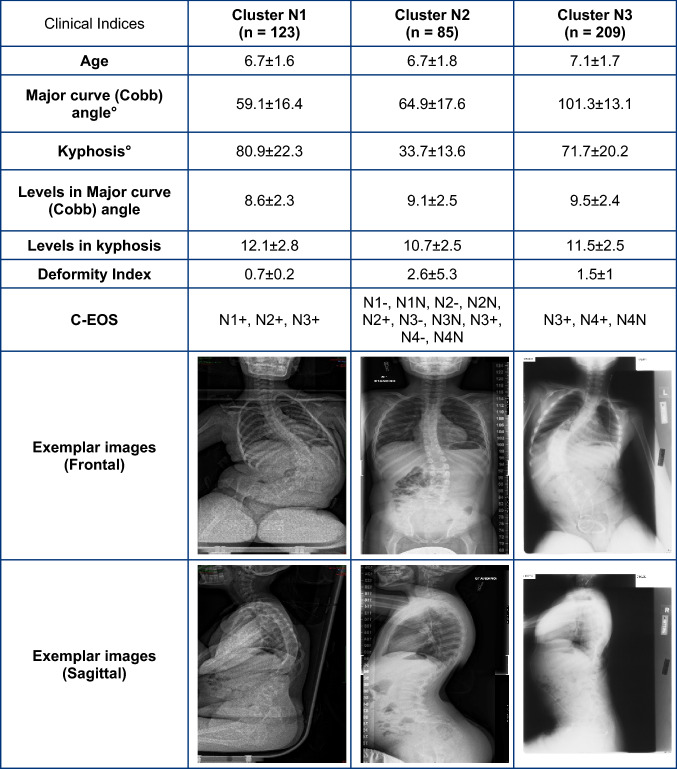
Average and standard deviations values of clinical indices along with exemplar radiographic images for Neuromuscular EOS clusters

**Table 4:**
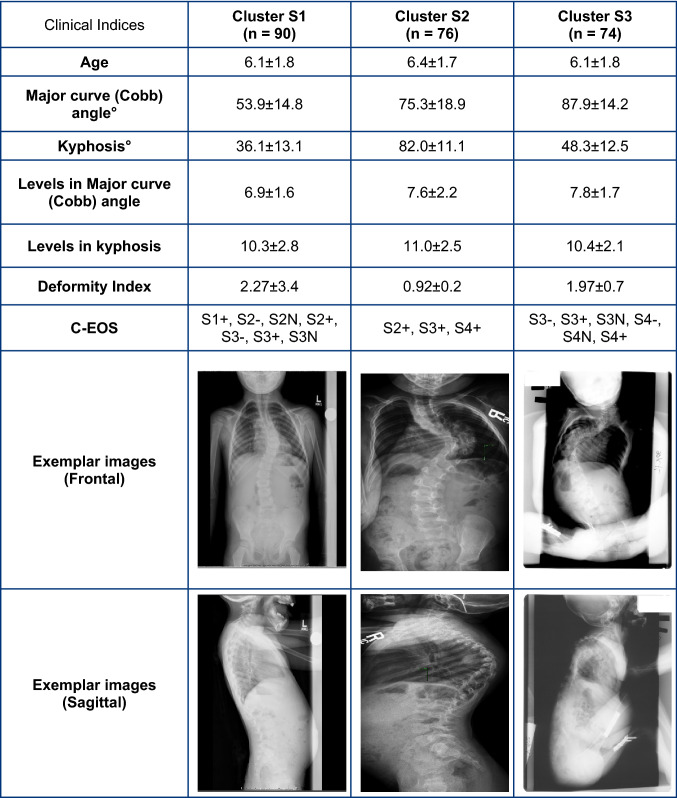
Average and standard deviations values of clinical indices along with exemplar radiographic images for Syndromic EOS clusters

## Discussion

This is the first study to perform automated fuzzy C-means clustering of EOS subjects based on pre-operative clinical indices, complementing the existing C-EOS classification system. While the C-EOS system uses Cobb angle and kyphosis, which were based on expert review informed by normative values derived from the literature, the current clustering method uses a data-driven approach, by using the highest average membership values to determine subject assignments to respective clusters, to create the subgroups. The smaller number (3) of subgroups per etiology generated by this clustering analysis could better focus research initiatives for the rare and heterogenous population of EOS patients to improve and standardize clinical decision-making. In contrast, as shown in Tables 1, 2, 3, 4, subjects in each cluster would be classified under multiple C-EOS groups which would not be conducive to clinical decision making [23]. This is supported by previous studies that have reported limitations of large EOS subgroups to aid in clinical outcome determination [10].

In this study, we identified unique clusters within each EOS etiology that were significantly different from each other based on all the clinical indices. Although one cluster in the idiopathic and congenital groups, respectively, had severe kyphotic deformities, these clusters still had a deformity index greater than one (i.e. greater major curve Cobb angle as compared to kyphosis). In contrast, both neuromuscular and syndromic groups had one cluster each with a deformity index less than one indicative of greater kyphosis as compared to the major curve Cobb angle. Although further validation is required for these methods to be widely adopted, the novel preliminary findings reported in the current study may point to differences in general deformity patterns observed between EOS etiologies, which are not immediately discernible with the C-EOS system. Additionally, while post-hoc testing showed significant differences across all clusters for all etiologies, we may not fully capture the heterogeneity as these are solely based on radiographic measurements.

Limited previous studies for automated clustering of patients with spine deformity have used techniques such as ISOData and K-means +  + [20, 24]. While these methods were able to create clusters based on patient deformity characteristics, they do not provide a quantitative basis to assign a patient to a particular cluster, and also lack the ability to assess whether or not a patient is an outlier. On the other hand, Fuzzy C-means, which is an automated unsupervised clustering algorithm that performs well on heterogenous datasets, uses membership values to provide a probabilistic estimate of a patient belonging to a particular cluster. Higher membership values (> 0.6) would indicate a greater probability of a patient’s assignment to a cluster, and thereby also help identify and exclude any outliers.

The current study is not without limitations. First, to better address the heterogeneity of EOS subjects, the current clustering method can be further improved. For example, a small number of subjects (*n* = 56 out of 1170), were excluded in any cluster due to low membership values (i.e. < 0.6) of the limited radiographic measurements. Inclusion of additional structural (such as thoracic deformity parameters), functional (such as pulmonary functional parameters) and time-based measures (Annual Progression Modifier (APM)) may help better address the variable presentations of EOS deformity. Secondly, we used a multi-institutional and multi-modal dataset with clinical indices measured made by multiple observers. Variations associated with such datasets would impact any classification method equally. However, the fuzzy C-means algorithm would be less affected by such variations due to the automated selection that is unbiased by preset cut-offs for clinical indices.

## Conclusion

An automated framework to cluster EOS patients based on pre-operative clinical indices was developed identifying three unique, data-driven subgroups for each C-EOS etiology category. Adoption of this clustering framework with further validation may assist with clinical applications such as surgical planning, optimization of intervention type and timing to improve clinical outcomes and reduce complications.

Link to the ACT-EOS web application: https://biomed.drexel.edu/labs/obl/toolkits/act-eos-application.

## Data Availability

All data are available with the Pediatric Spine Study Group.
